# The Different Clinical Courses of Legionnaires’ Disease in Newborns from the Same Maternity Hospital

**DOI:** 10.3390/medicina58091150

**Published:** 2022-08-24

**Authors:** Andrijana Kostic, Katarina Cukovic, Lidija Stankovic, Zorica Raskovic, Jelena Nestorovic, Dragana Savic, Aleksandra Simovic, Tijana Prodanovic, Suzana Zivojinovic, Sladjana Andrejevic, Ismihana Erovic, Zorana Djordjevic, Snezana Rsovac, Predrag Sazdanovic, Andjelka Stojkovic

**Affiliations:** 1 University Clinical Center, Clinic of Pediatrics, 34000 Kragujevac, Serbia; 2 Department of Pediatrics, Faculty of Medical Sciences, University of Kragujevac, 34000 Kragujevac, Serbia; 3 General Hospital, 84000 Bijelo Polje, Montenegro; 4 Department of Epidemiology, University Clinical Center, 34000 Kragujevac, Serbia; 5 University Children’s Clinic Tirsova, Pediatric and Neonatal Intensive Care, 11000 Belgrade, Serbia; 6 Department of Pediatrics, Faculty of Medicine, University of Belgrade, 11000 Belgrade, Serbia; 7 University Clinical Center, Clinic of Gynecology and Obstetrics, 34000 Kragujevac, Serbia; 8 Department of Anatomy, Faculty of Medical Sciences, University of Kragujevac, 11000 Belgrade, Serbia

**Keywords:** neonate, legionnaires’ disease, cysts, macrolides, quinolones

## Abstract

In children, the incidence of Legionnaires’ disease (LD) is unknown, hospital-acquired LD is associated with clinical risk factors and environmental risk, and children with cell-mediated immune deficiency are at high risk of infection. Both newborns were born in the same delivery room; stayed in the same hospital room where they were cared for, bathed, and breastfed; were male; were born on time, with normal birth weight, and with high Apgar score at birth; and survived this severe infection (*L. pneumophila*, serogroup 2-15) but with different clinical courses. In neonate 1, bleeding in the brain, thrombosis of deep pelvic veins, and necrosis of the lungs, which left behind cystic and cavernous changes in the lungs, were found, while neonate 2 suffered from pneumonia alone. The only difference in risk factors for LD between these two newborns is the number of days of illness until the start of azithromycin treatment (sixth versus the third day of illness). We suggest that a change in the guidelines for diagnosing and treating community-acquired pneumonia and hospital-acquired pneumonia in newborns is needed in terms of mandatory routine testing for *Legionella pneumophila*. Early initiation of macrolide therapy is crucial for the outcome of LD in the newborn.

## 1. Introduction

By searching the literature on the topic of intrahospital legionnaires’ disease (LD) in newborns, we obtained a few published papers. The data searched mainly described the fatal outcome in the newborns. The outcome of LD depends on the degree of contamination of the water reservoir, the intensity of exposure, and the sensitivity of the host after inhalation of the contaminated aerosol [1]. However, a study in 2016 indicated the possibility of transmission from person to person and that about 20% of LD is associated with travel [2,3]. The European Center for Disease Prevention and Control (ECDC) states in its leaflet that the key risk of LD is: “Wherever water droplets (aerosols) can form, there is a particular risk of infection at temperatures between 20 and 50 °C” [4].

Invasive lung infection caused by *Legionella pneumophila* (*L. pneumophila*) is associated with the inhalation of aerosol from water reservoirs and/or water systems where this bacterium multiplies in the protozoa of the biofilm formed from organic and inorganic substances [5]. The replication of *L. pneumophila* can inhibit an antibiotic with a high intracellular concentration [6]. If the replication of *L. pneumophila* is not stopped and does not suppress robust inflammation, acute and severe lung infection with necrosis of lung tissue in the form of massive nodular lesions marked by a “halo” sign or cavitation occurs [1,2,4,7]. The clinical picture of legionellosis ranges from invasive pulmonary and extrapulmonary infection (primary legionella infection is not in the lung) to Pontiac fever (nonspecific fever of the patient in epidemic conditions, self-limiting disease) [4,8,9,10]. 

Recently published data indicate a significant increase in the incidence of LD in adults over the past 27 years, in the United States and some European countries, but without a clear rationale [3,9,10,11]. In children, the incidence of LD is unknown, hospital-acquired LD is associated with clinical risk factors and environmental risk, and children with cell-mediated immune deficiency are at high risk of infection [12]. 

This manuscript aims to consider the difference in the clinical picture of two newborns to confirm the need for rapid, timely, accurate (“fluid”) diagnosis [7], and the importance of timely, targeted (“fluid”) treatment, to indicate the need for higher levels of attention on LD in children in the United States and Europe, to suggest revising of guidelines, and finally, to suggest additional research at the cellular and/or genetic level to determine the reason for differences in clinical courses between term infants, infected under the same conditions, with the same strain *L. pneumophila*.

Intrahospital infection with *Legionella pneumophila* (*L. pneumophila*) occurred in a small maternity hospital (where there is one delivery room, one room for newborns, and three rooms for mothers) and in the following circumstances: water tanks are supplied with water from a small local river, water is heated to a temperature of 40 °C (instead of 50–70 °C), and aerosol-generating devices were used (hospital water supply, hot water tanks, air conditioners, bathroom) for two weeks before the illness of newborns, in February 2022, which are, overall, favorable conditions for the reproduction of this atypical, Gram-negative, intracellular, facultative bacteria [13]. 

Both newborns were born in the same delivery room but not on the same day (with an interval of 8 days); they stayed in the same hospital room where they were cared for, bathed, and breastfed; both are male; both were born on time, with normal birth weight, with a high Apgar score at birth; both survived this severe infection but with different clinical courses (Table 1). Newborn 1 (NN1) was discharged from the maternity hospital on the 3rd day of life and stayed at home for 4 days, while newborn 2 (NN2) was discharged from the maternity hospital on the 3rd day of life and stayed at home for 3 days. 

NN1 was hospitalized at the Clinic of Pediatrics (CPUCC) on the 8th day of life. Four days later, newborn 2 (NN2) was hospitalized on day 6 of life. Serological confirmation that NN1 was infected with LD arrived on the day when NN2 was hospitalized, which accelerated the performance of diagnostic tests for LD for NN2 (Table 2). On the day of hospitalization of NN2, the local maternity hospital was closed and placed under the health supervision of the Ministry of Health of the Republic of Serbia. The main difference in the clinical pictures of these two newborns is that in addition to pneumonia with cystic formations in the lungs, consumption coagulopathy (DIC) developed in the form of sepsis, cerebral hemorrhage as well as thrombosis of the femoral vein and iliac vein, while NN2 suffered from pneumonia only. The family history of NN1 is burdened in the sense that the previous child died due to intestinal atresia in the postoperative course at 3.5 months of age, but personally, NN1 did not have any anomalies. The family history of NN2 is irrelevant.

**Figure 1:**
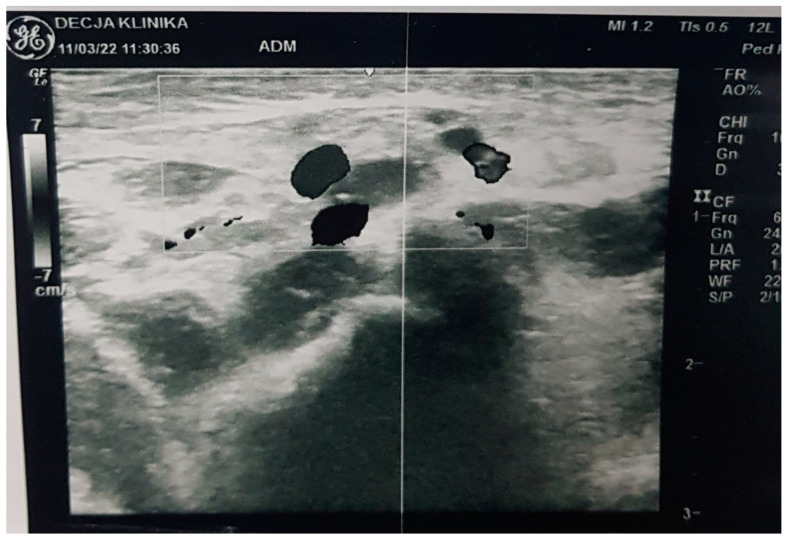
Ultrasound finding of blood vessels: The thrombosis of the femoral vein and iliac vein, on the right side, in newborn 1. The right femoral vein, right side, has a reduced diameter, long with 9 mm, lumen up to 50%, with a pre-obstructive dilatation of the blood vessel of about 5 mm. The right iliac external vein is from the level v. femoral along about 20 mm long filled with heteroechoic contents, more hyperechoic contents, corresponding to the thrombosis. A. femoris and a. iliaca external are preserved flow.

**Figure 2:**
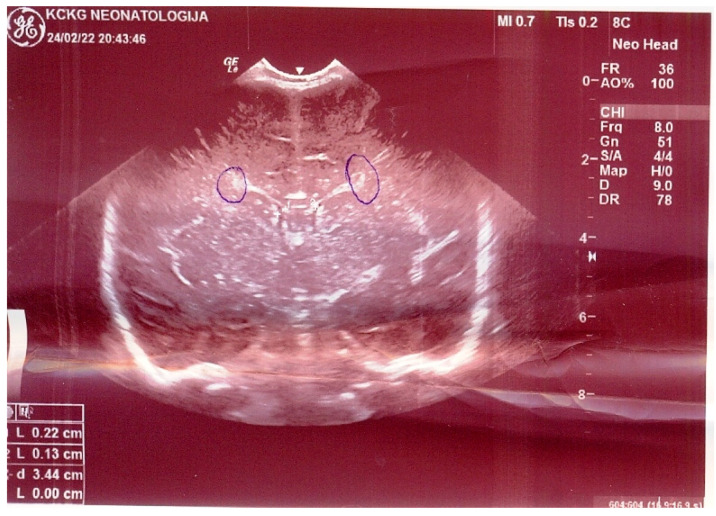
Ultrasound finding of the brain: The brain edema and on the right periventricular hyperechogenicity, the degree I, in newborn 1. Chambers easily asymmetric, VPRD = 2.2 mm, VPRL = 1.3 mm. Circular hyperechoic changes on both sides, around the tops of the lateral chambers. Right periventricular, hematoma with signs of resorption, 4 mm in diameter. Pronounced and diffuse hyperechogenicity in the parenchyma. Circulation in the a. pericalosa shows orderly velocities with low resistance RI = 0.53.

**Figure 3:**
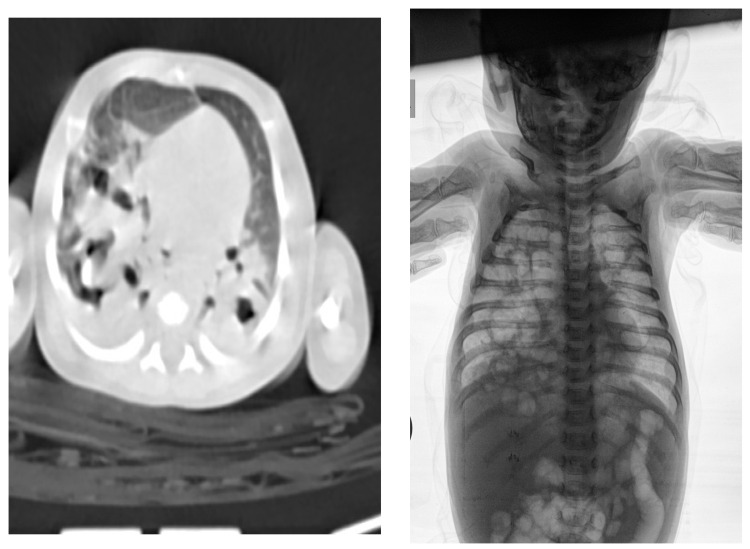
MSCT and X-ray of thoracic in neonate 1 indicate extensive, confluence, zones of lung parenchyma consolidation, on both sides, with signs of necrosis and formed thick wall cavities. The multislice computed tomography (MSCT) of the thoracic on the 17th day of hospitalization and X-ray of the thoracic on the 39th day of hospitalization shows cystic formations in the lungs. The MSCT finding on 17th day: The examination was performed natively and postcontrast, with axial sections 0.8 mm thick, with subsequent reconstructions in MPR and VRT. On both sides, diffuse in the lung parenchyma, large confluent zones of lung parenchyma consolidation can be seen, with hypodense zones of necrosis (density about 30 HU). Within the described consolidation zones, numerous thick-walled caverns can be seen, some of which communicate with each other, up to 13 mm in diameter. The anterior segments of the upper and lower lobes and the medial lingula segment of the right lung, as well as the anterior segment of the lower left lobe, are enveloped in aerations with smaller circular zones of consolidation. In the pleural cavities, on both sides, no free fluid is observed. The trachea, main, lobar, and segmental bronchi are preserved lumen and arborization. No significant mediastinal lymphadenopathy was observed. No pathological changes were seen in the bone structures of the chest wall shown. No pathological changes were seen in the sections through the upper abdomen. The X-ray finding on 39th day of hospitalization: Bilateral, diffuse in the pulmonary parenchyma of multiple oval cavitation zones with different wall thicknesses, which probably correspond to legionnaires’ disease. On the right are possible aero-liquid levels. Right hemidiaphragm of obscure contours.

**Figure 4:**
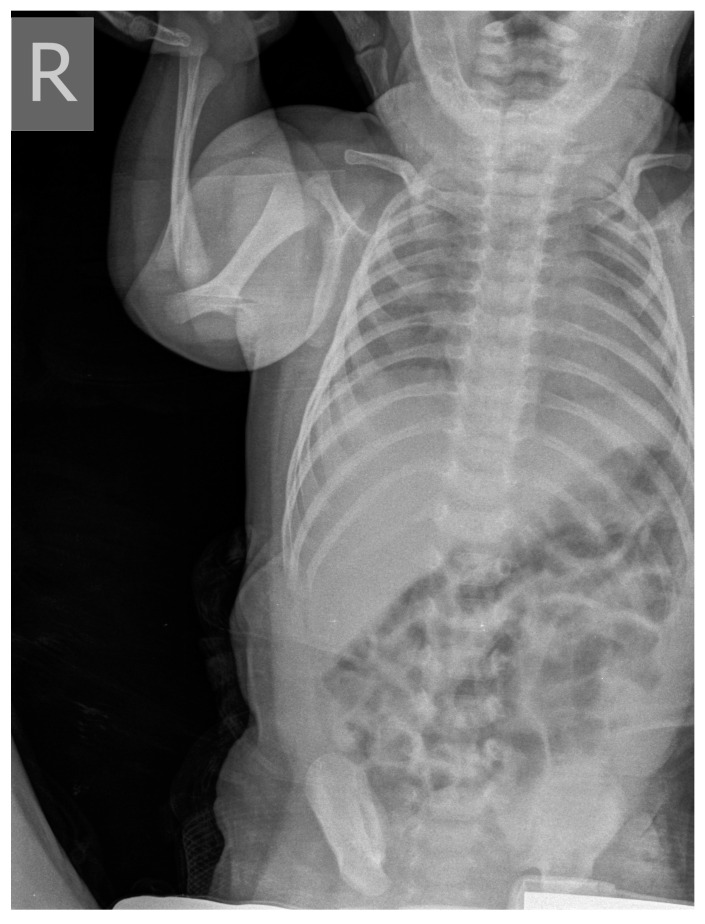
X-ray of thoracic in neonate 2 on the 33rd day of hospitalization: On the right, in the upper and medial lung fields, the transparency of the lung parenchyma is inhomogeneously reduced. On the left, the lower lung field is in superposition with heart shadow. The hemidiaphragms of clear contours.

NN1 was treated in the general hospital, on the 7th and 8th day of life, due to refusal of meals and drowsiness. The clinical picture of NN1 deteriorated during 24 h of hospital treatment in terms of cough, shortness of breath, moaning, sobbing, tachypnea, dyspnea, drop-in percutaneous oxygen saturation to 80%, with respiration rate 55/min, cardiac frequency 156/min. Then, auscultatory and respiratory sound over the lungs was diffusely attenuated, and all this took place despite treatment with oxygen therapy, systemic corticosteroid (sCS), and intravenous (iv) fluid replacement. Due to the described deterioration, NN1 was referred to the CPUCC. After 4 days, NN2 was referred to CPUCC, during the outpatient examination and without hospitalization at the local general hospital. The differences in the clinical picture of the two newborns on admission to the CPUCC are shown in Table 1. The results of hematological, biochemical, immunological, and microbiological analyses of both newborns are shown in Table 2. Although both newborns were infected with the same strain *L. pneumophila* (serogroup 2-15), consumption coagulopathy followed by severe clinical picture developed in NN1. Biomarkers of infection (C-reactive protein (CRP), procalcitonin (PCT), and interleukin 6 (IL 6)) were significantly elevated in NN1 as opposed to NN2 (Table 2). For NN1, ultrasound findings of femoral and iliac blood vessels, brain ultrasound, then lung radiography and chest scanner are shown in Figure 1, Figure 2 and Figure 3, while for NN2, lung radiography is shown in Figure 4. Serological tests of tracheal aspirate and urine confirmed the diagnosis of LD for both newborns (Table 2). NN1 was treated with macrolide from day 6 of the disease (azithromycin, then erythromycin) and ciprofloxacin (quinolone) from day 10 of the disease, while NN2 was treated with macrolide from day 3 of the disease (azithromycin, then erythromycin) and ciprofloxacin from day 11 of the disease.

For both neonates, tracheal aspirate samples were analyzed at Clinical Microbiology Department, University Clinical Center, Kragujevac, by Reverse Transcriptase Polymerase Chain Reaction (RT-PCR) testing (FilmArray^®^, Pneumonia Panel plus—IVD, Biofire, a Biomerieux company). At the same time, tracheal aspirate samples were also tested at The Department of Medical Microbiology, University Clinical Center of Serbia, Belgrade, by both RT-PCR (FilmArray^®^, Pneumonia Panel plus—IVD, Biofire, a Biomerieux company) and by the culturing on the Glycine Vancomycin Polymyxin Cycloheximide agar (GVPC) nutrient media. The urine was also examined for the presence of *L. pneumophila* antigens by a rapid immunochromatographic test (Uni-Gold legionella Urinary Antigen PLUS, trinity Biotech, Wicklow, Ireland). In the diagnosis of infectious agents that are difficult to cultivate in vitro or detected by direct examination, serological tests are used; i.e., to confirm LD, it is necessary to inoculate a sample of tracheal aspirate, sputum, bronchoscopic lavage, and blood on a special medium (buffered agar with yeast extract), while a non-invasive, antigenic test for *L. pneumophila* in urine is a sensitive method (80%) and specific (99%) for the rapid detection of the *L. pneumophila* serogroup 1 [12]. The whole genome nucleotide phylogeny of Legionella species, especially *L. pneumophila*, was not created because there are no microbiological laboratories in Serbia to create a phylogenetic tree of Legionella species, which is possible only in rare laboratories worldwide. 

## 2. Commentary

The question arises: does the outcome of LD depend only on the individual specific and nonspecific immune response of the newborn, especially the reaction of macrophages and T-cells, because, in our case, other risk factors for both newborns are identical [1,12]? It is still not clear why the clinical picture and clinical course of LD are not approximately the same in patients who are exposed to the same risk circumstances [8]? Many dilemmas are not clarified, although the consensus is reached for the effective treatment of *L. pneumophila* infection, in the sense of immediately administering an antibiotic with a high intracellular concentration, which is licensed for use in neonates (macrolide, rifampicin), or in the case that a newborn is vitally endangered, it would use an antibiotic that is not licensed for use in neonates (quinolone) with parental consent [12,14,15]. 

The clinical picture of pneumonia in newborns is similar regardless of whether it is caused by one of the most common microorganisms or a congenital infection (within 72 h of birth) or is caused by a virus, protozoa, tuberculosis bacillus, or a rare atypical bacterium [15]. Accordingly, pediatricians empirically choose a broad-spectrum antibiotic/s, taking into account the risk factors from the anamnesis and the initial results of the patient related to the diagnosis. However, if there is a rapid deterioration in the clinical picture despite empirical treatment of pneumonia, pediatricians consider some rare causes of pneumonia, including *L. pneumophila*, which is not covered by the Guidelines for Community-Acquired Pneumonia in neonates (CAP) [12,15]. Deterioration of the clinical picture, despite empirical treatment, is a circumstance related to delayed making an accurate diagnosis and delayed targeted choice of antibiotics, because additional time is needed to determine the microbiological cause. Any delay in macrolide and/or quinolone therapy for *L. pneumophila* pneumonia may increase the risk of severe complications [1,2,4,7]. 

The clinical picture of these two newborns is diametrically different, although they are infected with the same strain of *L. pneumophila*, serogroup 2-15; are in the same hospital circumstances; are of the same sex; have a high Apgar Score; are from term delivery, a vaginal route without instrumental intervention, normal body weight; and are on breastfeeding (Table 1). Unlike NN2, NN1 experienced a complication of pneumonia caused by *L. pneumophila* in terms of severe respiratory distress, respiratory failure (2nd day of illness), mechanical ventilation (4th to 24th day of illness), cerebral hemorrhage (2nd day of illness), severe anemia and thrombocytopenia (2nd day of illness), cystically altered lungs and right femoral and iliac vein thrombosis (18th day of illness). Cystic changes in the lungs of NN1 are shown on the X-ray of the lung and the multislice computed tomography (MSCT) (Figure 3). 

Cystic fibrosis, primary ciliary dyskinesia, and immunodeficiency were excluded by the monitoring of NN1 and additional diagnostics as possible reasons for a poor lung cleansing index and poor local immune defense mechanism with longer survival *L. pneumophila* in the respiratory system. 

The only difference in risk factors for LD observed between these two newborns is the number of days of illness until the start of treatment with azithromycin (sixth versus the third day of illness). To prove or disprove this hypothesis about the key start of macrolide treatment for LD outcome, we will consider the most important indicators of infection in these two newborns. 

Fever is a vital clinical sign and a sign of infection. Comparing this vital sign (Table 1) and the increase in inflammatory markers (CRP, PCT, IL6, d-dimer) (Table 2), a significant difference is observed between these two newborns, based on which we state the more severe clinical picture of NN1. The immune system’s defense against an infectious agent is based on the production of pyrogens that directly kill or inhibit the growth of the infectious agent, disrupt hypothalamic function, stimulate mobility, release white blood cells from bone marrow, and stimulate B-lymphocytes to produce antibodies. IL6 is a pyrogen that contributes to the body’s defenses by activating T-lymphocytes and NK cells, helping the lysis of pathogens within the cell, stimulating B-lymphocyte differentiation and proliferation and antibody production, stimulating bone marrow platelet release into the circulation and maturing megakaryocytes, contributing to megakaryocyte maturation and the permeability of blood vessels in inflammation via vascular endothelial growth factor, stimulating the synthesis of CRP and fibrinogen, and reducing the production of transferrin, fibronectin, and albumin [14]. A very high value of IL6 was found for NN1 in contrast to NN2, which confirms the severity of infection in NN1 (3335 vs. 760 pg/mL) (Table 2). 

D-dimer is a marker of sepsis in newborns and one of the end products of fibrin degradation under the influence of plasmin in the thrombus. The determination of serum d-dimer concentration is the test of choice in the emergency diagnosis of patients with suspected deep vein thrombosis (TDV), pulmonary embolism, disseminated intravascular coagulation (DIC), and malignancy, and it has a high negative predictive value [16]. During the DIC, the activation of procoagulant pathways is abnormally increased; i.e., the level of blood clotting proteins is reduced, which results in bleeding, thrombosis, and embolism [16]. In NN1, bleeding in the brain was found (Figure 2), as was deep vein thrombosis (TDV) (Figure 1) and lung necrosis, which left behind cystic and cavernous changes in the lungs (Figure 3). D-dimer values for both neonates were elevated almost equally (Table 2). Biomarkers of infection were elevated for both neonates, but they were extremely high for NN1, which confirms the severe clinical picture of LD with the listed complications in NN1 (Table 2). Periventricular hematoma in the brain with signs of resorption is also seen in newborns who do not suffer from LD, so, in this case, the ultrasound finding of the brain for NN1 is not pathognomonic. 

Hypovitaminosis D is observed in both newborns, which confirms their immune susceptibility to respiratory infections (Table 2) [17]. An increase in troponin-I was found in both neonates as evidence of transient myocardial ischemia but with normal ultrasound findings of the heart (Table 2) [18]. 

The diagnosis of LD was confirmed by serological tests of tracheal aspirate and urine for NN1 on day 4 of the disease (Table 2), but due to erythromycin and rifampicin scarceness, targeted treatment was started on day 6 of azithromycin iv by day 10 diseases. From the 10th day of the disease in NN1, treatment was carried out with erythromycin iv and ciprofloxacin iv for 14 days. In addition to this, from the first day of hospitalization, the treatment was carried out with the following drugs: antibiotics according to the guidelines for CAP (empirical choice and according to the antibiogram for isolated germs), oxygen therapy, mechanical ventilation, sCS (methylprednisolone, 1–35th day of illness), surfactant (poractant alfa in 2 doses), theophylline, diuretic, antiepileptic (phenobarbital), sedative (midazolam), proton pump inhibitor (pantoprazole), low-molecular-weight heparin (NMH) nadroparin-calcium during 33 days of illness, immunoglobulin G iv, transfusion of deplasmated erythrocytes, transfusion of concentrated platelets and probiotic. We will compare the course of treatment between NN1 and NN2. NN2 was treated with azithromycin from day 3 of the disease for 3 days, then erythromycin for 14 days, ciprofloxacin from day 11 of the disease for 10 days, and it had a favorable course of the disease and without complications, unlike NN1. The clinical picture of NN1 and NN2 necessitated quinolone treatment after the administration of azithromycin. The severity of the clinical picture of both newborns caused a difference in the length of hospitalization: 49 days for NN1 compared to 21 days for NN2. 

Azithromycin has anti-inflammatory, immunomodulatory, and antimicrobial action and is particularly important in the treatment of severe respiratory infections resulting from poor regulation of the inflammatory cascade as in LD [5,6,8,15]. *L. pneumophila* inhibits the metabolism of human macrophages by inhibiting numerous pattern recognition receptors (PPRs), inhibiting protein translation in the host macrophage, and establishing an upper regulation of interleukin-1-alpha, resulting in an increased production of proinflammatory cytokines by uninfected cells (observers) competent for translation. Then, an effector-mediated host protein translation restriction is established, aerobic glycolysis occurs, and overall, *L. pneumophila* avoids host defense and at the same time encourages the formation of a special phagosome that allows bacterial replication to continue [5]. The replication of *L. pneumophila* is strongly inhibited by an antibiotic with a high intracellular concentration, bearing in mind that azithromycin achieves 5–10 times higher concentration in the macrophage than erythromycin [6,19]. 

Systemic corticosteroid (sCS) is a drug commonly used in sick newborns mainly to prevent the development or treat bronchopulmonary dysplasia, despite the warning that sCS significantly slows the development of cognitive and motor functions [20]. In addition, sCS is known to interfere with T-lymphocyte and macrophage function, which increases the risk of spreading and deepening infection [12,15]. On the other hand, the desired effects of corticosteroids (sCS, inhaled CS) are necessary for the treatment of severe broncho-obstruction in newborns, along with other drugs (inhaled bronchodilator, theophylline, azithromycin) [19]. The length of application of sCS or ICS in the newborn is not always defined for a particular disease/condition but is individual and according to the pediatrician’s assessment, taking into account the wishes and adverse effects [19,20,21]. It cannot be ignored that sCS was administered in NN1 for 35 days and that cystic and cavernous changes in the lungs and pelvic TDV occurred during this period, which means that in this case, the desired anti-inflammatory effect of sCS was not achieved [13,20,21]. Unlike NN1, NN2 was treated with cCS for 10 days, ICS for 6 days, with oxygen therapy, an inhaled bronchodilator, listed antibiotics, and NMH (3 days).

## 3. Conclusions

Respiratory infection *L. pneumophila* is an increasingly common challenge for pediatricians. Therefore, it is necessary to change the guidelines for diagnosing and treating CAP and hospital-acquired pneumonia in newborns in terms of mandatory routine testing on *L. pneumophila*. Consequently, the recommendation for empirical antibiotic choice in neonates with CAP should be changed in terms of positioning Azithromycin as the first choice for LD and ciprofloxacin as a reserve antibiotic for life-threatening LD, thus implementing “timely and targeted” LD therapy in newborns, and we are not alone in this conclusion [22]. Corticosteroids, with their anti-inflammatory effect, did not prevent the development of cysts and caverns in the lungs or deep vein thrombosis in the pelvis of NN1, so their use in respiratory failure of newborns, peculiarly, should be specified in the future. It is clear that additional research is needed at the cellular, immune and genetic levels (including the creation of a phylogenetic tree based on the nucleotide sequences of the genetic material of *L. pneumophila*) regarding the predisposition of the newborn to a severe clinical picture during atypical *L. pneumophila* infection not only for serogroup 1 but also for 2–15. With this work, we raise the level of attention to LD in all children in the future.

## Figures and Tables

**Table 1:** Relevant clinical features of neonates at admission.

	NN1, Boy	NN2, Boy
Apgar score at 1st/5th minute	10/10	9/10
Weight at birth (g)	4050	3850
Birth naturally way	yes	yes
Breastfeeding, exclusively	yes	yes
Family history	the older boy died of congenital intestinal atresia	-
Age of hospitalization (day)	8	6
Duration of 1st hospitalization (day)	49	21
Clinical picture at admission in Pediatric Clinic UCC	NN1 had a preserved sensorium, was high febrile 39.4 °C (rectal), with tachy-dyspnea, sobs, and moans, indents jugulum, dissatisfied cries, TM 4050 g, subclinical jaundice of the skin, and visible mucosa. Auscultation revealed attenuated respiratory sound, diffusely fine crackles, SaO2 82%, R 56/min, F 196/min. The umbilical stump persisted, the surrounding skin became red and swollen, there was hypotonia of the body axis, large fontanelle within the bony borders, greatness 20 × 30 mm. The other physical findings were normal.	NN2 had the preserved sensorium, was afebrile 37.7 °C (rectal), TM 3920 g, eupnoeic, presented sobs and moans, plethoric and icteric skin, nasal vestibules filled with seromucous secretion, and hyperemic throat. Auscultatory revealed a normal breathing sound is heard with transmitted wheezes from the upper parts of the airways and systolic murmur of 1-2/6 according to Levin, SaO2 97%, R 32/min, F 168/min. The umbilical stump persisted, thin, and the borders developed a serous–hemorrhagic discharge. There was mild hypotonia of the shoulder girdle and trunk axis, primitive reflexes were slowly elicited, large fontanelle was below the plane of the bony borders, and slightly spaced sutures, greatness 40 × 40 mm. The other physical findings were normal.

SaO_2_, per skin saturation of oxygen; R, respiration rate; F, heart rate; LF, large fontanelle.

**Table 2:** Relevant hematological, biochemical, immunological, and microbiological results.

		NN1, Boy	NN2, Boy
Infection related biomarkers	CRP (mg/L)	219, 223, 255, 209, 127, 14, 62, 7	26, 69, 5.6, 0.5, 0.2
	PCT (ng/mL)	83, 40, 76, 92, 1.0, 0.8, 0.7, 0.2, 0.1, 0.1	0.67, 1.14, 0.08, 0.13
	IL6 (pg/mL)	3335, 94.4	760
WBC (×10/−9 L)		11.7, 3.0, 2.5, 4.7, 44, 20.2, 19.1, 21, 17	31.6, 24.4, 19.2, 24.1, 12.8, 11.7, 12.7
n (%)		72, 58, 37, 18, 19, 66, 70, 65, 58, 45	56, 58, 63, 33, 36, 44
RBC (×10/−12 L)		3.7, 3.5, 3.7, 3.0, 3.5, 3.9, 4.3, 3.9, 4.9, 3.3, 4.1, 2.8, 3.1	4.0, 4.2, 4.1, 3.7, 3.4, 3.7
hemoglobin (g/L)		126, 114, 100, 119, 125, 125, 157,103, 86, 91	143, 114, 136, 143, 117, 107, 110
Tr (×10/−9 L)		435, 441, 99, 53, 40, 199, 211, 616, 600, 591	374, 437, 266, 705, 367, 611, 465
PT (s)		21.9	15.2, 13.3, 14.8
PT(INR)		1.7	1.13, 1.03, 0.99, 1.11
APTT (s)		41.2	34.3, 27.5, 28.8, 35
d-dimer (μg/mL FEU)		6.3, 8.8, 7.0, 1.9, 3.3, 2.1	1.99, 12.6, 2.9, 1.2, 0.8, 0.8
Fibrinogen (g/L)		7.4, 4.1, 4.4	7.3, 3.3, 2.4
Anti Xa 12 h (IU/mL)		0.35, 0.41	-
Feritin (μg/L)		920	791
Liver function test	AST (IU/L)	36, 42, 43	33, 39, 42
	ALT (IU/L)	28, 18, 22	28, 97, 53
	ALP (IU/L)	79	-
	Gamma GT (IU/L)	35, 242, 102, 293	33
	Serum bilirubin total/direct (μmol/L)	142/18	127/11, 26/8
	Serum total protein/albumin(g/L)	64/25, 63/35	57/33
Urea (mmol/L)		3.7, 4.3	3.6, 4.0, 4.9
Creatinine (μmol/L)		56, 48	54, 32, 30
Troponin (mcg/L)		0.013	0.015, 0.07, 0.06, 0.13
Gas analysis -capillary	pH	7.3, 7.2, 7.5, 7.6, 7.4, 7.4	7.4, 7.4, 7.4, 7.4, 7.5
	pCO2 (kPa)	6.8, 10, 4.1, 3.6, 5.3, 5.3	4.1, 3.6, 5.5, 5.1, 4.8
	pO2 (kPa)	9.5, 6.4, 5.8, 6.0, 9.5, 7.7	18.0, 12.1, 4.9, 3.6, 8.9
	K (mmol/L)	2.9, 3.8, 2.3, 3.0, 5.1, 5.6	4.5, 4.6, 5.7, 4.8, 4.9
	Na (mmol/L)	135, 132, 129, 136, 134, 136	132, 134, 137, 128, 130
	HCO3 std	21, 25, 26, 31, 23, 23	22, 22, 26, 25, 27
	Glu (mmol/L)	11, 6.5, 9.5, 6.7, 5.0, 5.3	5.3, 5.0, 5.1, 4.8, 5.0
	Lac (mmol/L)	4.1, 4.2, 3.6, 2.4, 1.4, 1.8	2.5, 3.6, 1.9, 1.6, 2.3
	BEecf (mmol/L)	−4.2, 0.8, 4.9, 7.3, −2.2, −2.8	−5.0, −5.9, 2.1, 0.9, 3.3
Vitamin D (ng/mL)		11.7	16.2
Serology/microbiology findings	PCR-SARSCoV2	negative	negative
	Blood culture I	coagulase-negative staphylococci	coagulase-negative staphylococci
	Blood culture II	sterile	sterile
	Tracheal aspirate by RT-PCR testing	*Legionella pneumophila* serogroup 2-15	*Legionella pneumophila* serogroup 2-15
	Culture of tracheal aspirate on the GVPC nutrient media	*Legionella pneumophila*, *Pseudomanas aeriginosa*	*Legionella pneumophila*
	Culture of navel swab	coagulase-negative Staphylococci	Klebsiella, Enterobacter spp.
	Urine by rapid Uni-Gold plus	without pathological flora	without pathological flora
	TORCH screen	not detected	not detected
Cells immuno-phenotyping (after RBC lysis)		A neutrophil population of 76% and 14% of lymphocytes stood out. In the lymphocyte population, a lower percentage of T lymphocytes is registered, while 19% are B lymphocytes.	-

NN, neonate; CRP, C-reactive protein; PCT, procalcitonin; WBC, leukocytes; n, neutrophils; RBC, erytrocytes; Tr, platelets; LDH, lactic dehydrogenase; ALT, alanine aminotransferase, AST, aspartate aminotransferase, ALP, serum alkaline phosphatase, IL6, interleukin 6; Gamma Gt, gamma-glutamyl transferase; PCR, polymerase chain reaction and detection of the bacterial genome by the molecular method; Coag.nSt, coagulase-negative staphylococci; S. aureus, Staphylococcus aureus; anti-Xa, anti-factor Xa activity; RT-PCR, FilmArray^®^, Pneumonia Panel plus-IVD, Biofire, a Biomerieux company; GVPC, Glycine Vancomycin Polymyxin Cycloheximide agar; rapid Uni-Gold plus, immunochromatographic test (Uni-Gold legionella Urinary Antigen PLUS, trinity Biotech, Wicklow, Ireland); TORCH screen, a group of blood tests for toxoplasmosis, rubella cytomegalovirus, herpes simplex, and HIV; Note: C-reactive protein (CRP) and procalcitonin (PCT) were performed in parallel at the admission of both newborns, but later during treatment, this was not completed. The dynamics of analyzing CRP and PCT depended on the deterioration, the severity of the newborn’s clinical picture, the value of other biochemical analyzes, microbiological results and clinical and biochemical response to the applied therapy in a certain period of treatment.

## Data Availability

Data sharing not applicable.
